# Urinary growth differentiation factor 15 predicts renal function decline in diabetic kidney disease

**DOI:** 10.1038/s41598-023-39657-7

**Published:** 2023-08-02

**Authors:** Toma Oshita, Shun Watanabe, Takafumi Toyohara, Ryota Kujirai, Koichi Kikuchi, Takehiro Suzuki, Chitose Suzuki, Yotaro Matsumoto, Jun Wada, Yoshihisa Tomioka, Tetsuhiro Tanaka, Takaaki Abe

**Affiliations:** 1 Division of Nephrology, Endocrinology, and Vascular Medicine, Tohoku University Graduate School of Medicine, Sendai, Japan; 2 Division of Medical Science, Tohoku University Graduate School of Biomedical Engineering, Sendai, Japan; 3 Laboratory of Oncology, Pharmacy Practice and Sciences, Tohoku University Graduate School of Pharmaceutical Sciences, Sendai, Japan; 4 Department of Nephrology, Rheumatology, Endocrinology and Metabolism, Okayama University Graduate School of Medicine, Dentistry and Pharmaceutical Sciences, Okayama, Japan; 5 Department of Clinical Biology and Hormonal Regulation, Tohoku University Graduate School of Medicine, Sendai, 980-8574 Japan

**Keywords:** Chronic kidney disease, Predictive markers

## Abstract

Sensitive biomarkers can enhance the diagnosis, prognosis, and surveillance of chronic kidney disease (CKD), such as diabetic kidney disease (DKD). Plasma growth differentiation factor 15 (GDF15) levels are a novel biomarker for mitochondria-associated diseases; however, it may not be a useful indicator for CKD as its levels increase with declining renal function. This study explores urinary GDF15’s potential as a marker for CKD. The plasma and urinary GDF15 as well as 15 uremic toxins were measured in 103 patients with CKD. The relationship between the urinary GDF15-creatinine ratio and the uremic toxins and other clinical characteristics was investigated. Urinary GDF15-creatinine ratios were less related to renal function and uremic toxin levels compared to plasma GDF15. Additionally, the ratios were significantly higher in patients with CKD patients with diabetes (*p* = 0.0012) and reduced with statin treatment. In a different retrospective DKD cohort study (U-CARE, n = 342), multiple and logistic regression analyses revealed that the baseline urinary GDF15-creatinine ratios predicted a decline in estimated glomerular filtration rate (eGFR) over 2 years. Compared to the plasma GDF15 level, the urinary GDF15-creatinine ratio is less dependent on renal function and sensitively fluctuates with diabetes and statin treatment. It may serve as a good prognostic marker for renal function decline in patients with DKD similar to the urine albumin-creatinine ratio.

## Introduction

Diabetic kidney disease (DKD) is one of the main causes of CKD and results in end-stage kidney disease if not treated properly. For a long time, the estimated glomerular filtration rate (eGFR) and urine albumin-creatinine ratio (UACR) were used as the main indicators for the progression and outcomes of DKD^1^. Because several reports showed that the UACR is not sensitive enough as a marker in the early stages of DKD, other biomarkers have been investigated^2,3^. Although some protein biomarkers such as ANGPTL4 and MCP-1 have been identified, none of the other biomarkers have shown a prognostic value greater than UACR^4^.

We recently showed that plasma growth differentiation factor 15 (GDF15), a member of the transforming growth factor-β (TGF-β) superfamily, is a diagnostic marker for mitochondrial diseases^5^. In other disorders caused by mitochondrial dysfunction such as diabetes^6^, senescence^7^, cardiovascular disease, neurodegenerative disease^8^, and COVID-19^9^, the plasma GDF15 levels are increased. However, as for CKD, plasma GDF15 might not serve as a useful prognostic or diagnostic marker as recent reports have shown that plasma GDF15 levels are inversely correlated with eGFR^10,11^. On the other hand, only a few studies have investigated the relationship between urinary GDF15 and other parameters in kidney diseases^12^, although it could be a potential diagnostic tool for kidney diseases.

This study investigated the relationship between the urinary GDF15-creatinine ratio and other uremic toxins and clinical characteristics, which highlighted the difference between urinary GDF15 and plasma GDF15. We also explored the association between the urinary GDF15-creatinine ratio and DKD. A further retrospective cohort study suggested that the urinary GDF15 -creatinine ratio could be a good prognostic factor for the decline of kidney function in DKD, similar to UACR.

## Materials and methods

### Recruitment of patients with chronic kidney disease

We recruited 103 patients with CKD who were treated at the outpatient clinic in Tohoku University Hospital. In the cohort treated with a statin, 39 patients with CKD at Tohoku University Hospital were recruited. The study protocols were approved by the ethics committee of Tohoku University (2022-1-823, 2012-3-19) and complied with the guidelines of the Declaration of Helsinki. All patients signed written informed consent.

### Quantification of growth differentiation factor 15 and uremic toxins

The concentrations of GDF15 in the plasma and urine samples were measured using a Quantikine Human GDF15 ELISA Kit (R&D Systems, Minneapolis), as previously described^13^. The urinary GDF15 was normalized by the urinary creatinine (urinary GDF15 -creatinine ratio). The levels of microbiota-derived uremic toxins, including triethylamine-*N*-oxide (TMAO), phenol sulfate (PS), *p*-cresol sulfate (pCS), and indoxyl sulfate (InS) were measured by LC/MS/MS^14^. 1-methyladenosine (m1A) was also measured as a marker for kidney damage^15^. In the measurement, deuterated internal standards were added for all analytics. The measurements were performed using a TSQ Quantum Ultra triple quadrupole mass spectrometer (Thermo Fisher Scientific, Waltham) equipped with a heated electrospray ionization source system. Samples were scrutinized in a single reaction monitoring mode by monitoring the ion transitions. Data were acquired and analyzed using Xcalibur™ software, version 2.1 (Thermo Scientific, Waltham).

### Recruitment and measurement of the U-CARE cohort

The urinary biomarker for the continuous and rapid progression of diabetic nephropathy (U- CARE) study was a multicentre, observatory clinical study that aimed to interrogate the urinary biomarkers in diabetic nephropathy (UMIN 00011525)^16^. We recruited 342 patients for this study as reported previously^14^ and analyzed the levels of GDF15 in the plasma and urine samples. The analyses were approved by the ethics committees of both Tohoku University and Okayama University (approval number: 1702-026). All patients provided written informed consent.

### Statistical analysis

To identify the urinary GDF15 levels that are independently associated with the decline in 2-year eGFR, three multiple regression models were used, as previously described^14^. Briefly, we built three models as an independent variable examined by a variance inflation factor (VIF) < 10, the urinary GDF15 level normalized by urinary creatinine, and the decrease in eGFR over 2 years, which were used as objective variables to perform the multiple regression analysis. Model 1 was the crude model. Model 2 was adjusted for known factors: age, sex, body mass index (BMI), systolic blood pressure (SBP), HbA1c, and log (eGFR), all of which were baseline values. Model 3 was the full model, adjusted for the Model 2 factors plus other clinical factors, such as diastolic blood pressure (DBP), alanine aminotransferase (ALT), total cholesterol (TC), triglyceride (TG), high-density lipoprotein (HDL), and uric acid (UA).

A logistic regression analysis was used to identify factors independently associated with the decline in eGFR during the 2 years. In this study, we defined a 15% reduction in eGFR during the 2 years as the decline in eGFR. This criterion was based on previous research^17^ in which at least a 15% reduction in eGFR would be associated with a subsequent risk of end-stage renal failure. Using this definition, 32 patients in the U-CARE study had a 15% reduction in eGFR; thus, we enrolled enough patients for statistical analyses. To identify the factors independently associated with the decline in eGFR during the 2 years, the same three multiple logistic regression models were used, as previously reported^14^: Model 1, only log (urinary GDF15) and UACR, which is a known predictive factor for DKD^18^ (crude model); Model 2 and Model 1 with known factors (age, gender, BMI, SBP, HbA1c, and log(eGFR))^19^; and Model 3 and Model 2 with other factors (duration, DBP, ALT, TC, TG, HDL, and UA). The results are shown as odds ratios (ORs) with 95% confidence intervals (CIs). ORs for all continuous variables were computed for each standard deviation (SD) change. The stepwise method was performed according to Akaike's information criterion. Other statistical analyses were performed using Graph Pad Prism version 9 (GraphPad Software, San Diego, California, USA, http://www.graphpad.com). Statistical significance was set at *p* < 0.05.

## Results

### Correlation of plasma and urinary GDF15 with kidney function and uremic toxins

In CKD patients, various uremic toxins accumulate depending on the intensity of renal failure and damaged tissues^20,21^. It has been reported that the microbiota-derived uremic toxin plays a role in the pathogenesis of CKD^14^; however, the extent of the correlation between the GDF15 levels and the concentration of microbiota-derived uremic toxins has not been well investigated. To investigate the differences between plasma and urinary GDF15 in CKD, we enrolled 103 CKD patients (Table 1). The mean age of the participants was 69.2 ± 12.3 years, and 62 participants (60.2%) were male. The majority of the patients had hypertension (90.3%); however, the blood pressures were well controlled. Diabetes and dyslipidemia were being treated in some patients (44.7% and 31.1%, respectively).

**Table 1:** Baseline characteristics of 103 patients with chronic kidney disease.

Number of patients	103
Demographics	
Age, y	69.2 ± 12.3
Gender, male, n (%)	62 (60.2)
BMI, kg/m^2^	25.2 ± 4.4
Medical history	
Hypertention, n (%)	93 (90.3)
Diabetes mellitus, n (%)	46 (44.7)
Hyperlipidemia, n (%)	32 (31.1)
Medications	
Antihypertensives, n (%)	82 (79.6)
ACEi/ARB, n (%)	66 (64.1)
Hypoglycemics, n (%)	32 (31.1)
Metoformine, n (%)	13 (12.6)
SGLT2i, n (%)	14 (13.6)
DPP4i, n (%)	23 (22.3)
Statin, n (%)	44 (42.7)
Clinical features	
sBP, mmHg	128 ± 8.7
dBP, mmHg	73 ± 6.3
Serum creatinine, mg/dL	1.33 ± 0.86
eGFR, mL/min/1.73 m^2^	60.3 ± 24.8
GDF15 concentration	
Plasma GDF15, pg/mL, median (IQR)	1466.7 (1002.1–2005.2)
Urinary GDF15, ng/mmolCre, median (IQR)	1180.0 (683.9–1851.9)

BMI, body mass index; ACEi, angiotensin converting enzyme inhibitor; ARB, angiotensin II receptor blocker; SBP, systolic blood pressure; eGFR, estimated glomerular filtration rate; DBP, diastolic blood pressure.

**Figure 1:**
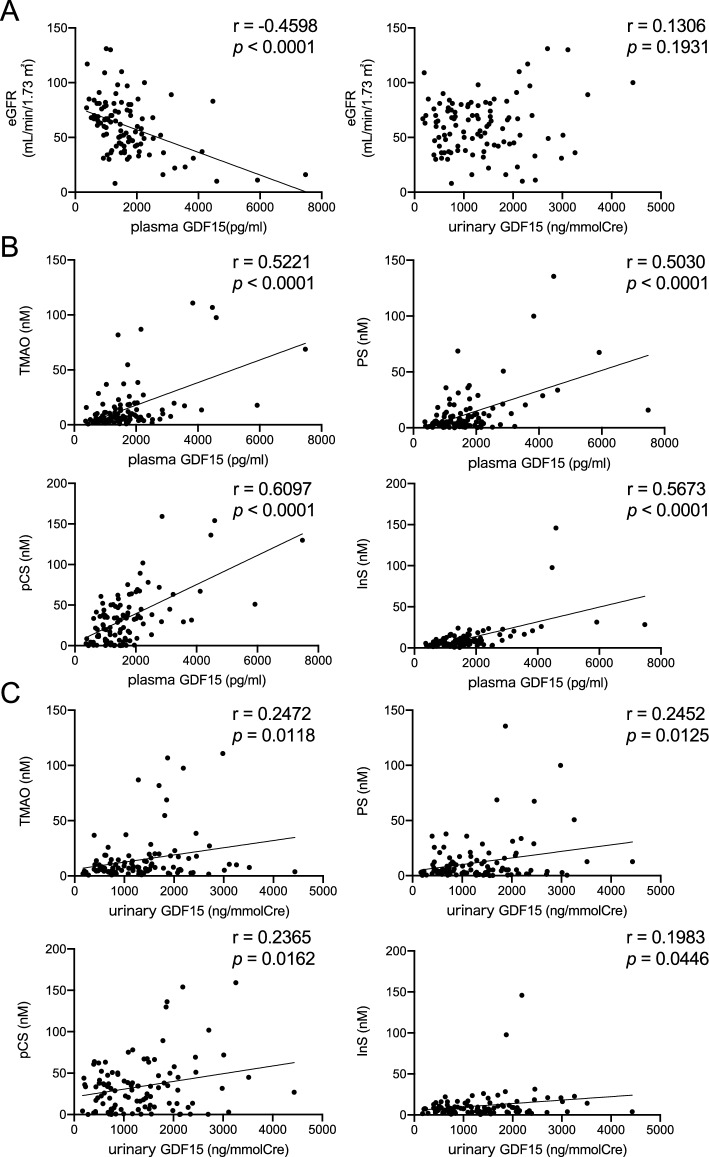
Correlations of plasma and urinary GDF15 with renal function and uremic toxins. Correlations of plasma and urinary GDF15 with eGFR (**A**); correlation of microbiota-derived uremic toxins, including triethylamine *N*-oxide (TMAO), phenol sulfate (PS), *p*-cresol sulfate (pCS), and indoxyl sulfate (InS), with plasma GDF15 (**B**) and urinary GDF15 (**C**). Spearman correlation coefficient was used to assess the relationships. *p* < 0.05 was considered statistically significant. GDF15, growth differentiation factor 15.

In the relationship between GDF15 and eGFR, plasma GDF15 was inversely correlated with eGFR (r =  − 0.4598, *p* < 0.0001), whereas the urinary GDF15 to creatinine ratio was not correlated with eGFR (r =  − 0.1306, *p* = 0.1931) (Fig. 1A). Next, we analyzed the correlations between GDF15 and microbiota-derived uremic toxins in the serum, such as TMAO, PS, pCS, and InS, all of which increase with CKD progression^14^. The plasma GDF15 was highly correlated with the uremic toxins (TMAO: r = 0.5221, *p* < 0.0001; PS: r = 0.5030, *p* < 0.0001; pCS: r = 0.6097, *p* < 0.0001; InS: r = 0.5673, *p* < 0.0001) (Fig. 1B). On the other hand, the urinary GDF15 to creatinine ratio was less correlated with the microbiota-derived uremic toxins (TMAO: r = 0.2472, *p* = 0.0118; PS: r = 0.2452, *p* = 0.0125; pCS: r = 0.2365, *p* = 0.0162; InS: r = 0.1983, *p* = 0.0446) compared to plasma GDF15 (Fig. 1C). Similar to the microbiota-derived uremic toxins, the urinary GDF15 to creatinine ratios were less correlated with the uremic marker increased in cellular damages, m1A, when compared to the plasma GDF15 levels^15^ (Fig. S1). These data indicated that urinary GDF15 would be less correlated with eGFR and uremic toxins, while plasma GDF15 is well correlated.

### Urinary GDF15 levels increased in CKD patients with diabetes mellitus

**Figure 2:**
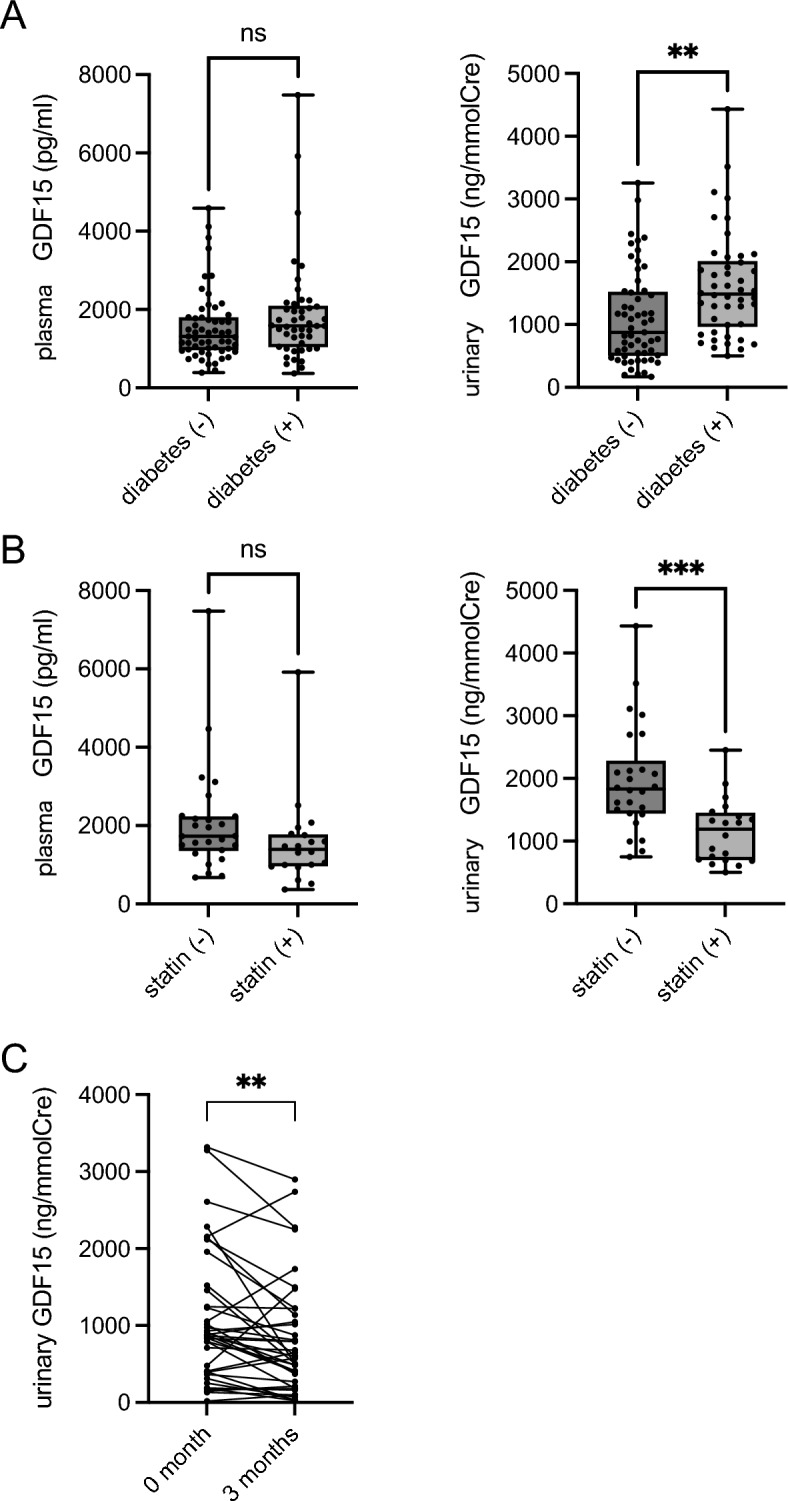
The differences of dynamics in the diabetic state between plasma and urinary GDF15. (**A**) Concentration of plasma and urinary GDF15 in patients with and without diabetic kidney disease (DKD) (n = 46 and 57, respectively), (**B**) concentration of plasma and urinary GDF15 in patients with DKD receiving statin treatment or not (n = 20 and 26, respectively). (**C**) concentration of urinary GDF15 in CKD patients treated with statin (n = 39). An unpaired t-test (**A**,**B**) and Wilcoxon matched-pairs signed rank test (**C**) were used to compare two groups. Data represent mean ± SD, **p < 0.01, ***p < 0.001. GDF15, growth differentiation factor 15.

We next explored the association of plasma and urinary GDF15 with diabetes mellitus because previous studies have shown that plasma GDF15 is involved in diabetic progression^6^. The urinary GDF15 to creatinine ratio was notably increased in patients with diabetes (non-diabetic vs. diabetic: 1100 ng/mmol vs. 1616 ng/mmol Creatine, *p* = 0.0012). However, plasma GDF15 was not significantly associated with diabetes (non-diabetic vs. diabetic: 1550 pg/mL vs. 1855 pg/mL, *p* = 0.1632) (Fig. 2A, eGFR was not significantly different between non-diabetic patients and diabetic patients: 58.7 mL/min/1.73 m^2^ vs. 62.2 mL/min/1.73 m^2^, *p* = 0.2223). These data suggested that urinary GDF15 might reflect renal mitochondrial dysfunction more sensitively than plasma GDF15. Since some medications, including statins, have been known to protect the kidneys against diabetes^22^, we further investigated the effect of statins on the plasma and urinary GDF15 levels in diabetic patients. As a result, the urinary GDF15 to creatinine ratio was significantly lower in the statin-treated group compared to the non-statin group (non-statin vs. statin: 1971 vs. 1154 ng/mmol creatinine, *p* = 0.0005), while plasma GDF15 was not significantly different (Fig. 2B). Contrarily, the plasma and urinary GDF15 levels in non-diabetic patients were not significantly different despite being on statin treatment (Fig. S2). We further explored the effect of statin treatment on the urinary GDF15 to creatinine ratio (the CKD patient profile is shown in Table S1). The urinary GDF15 to creatinine ratio was significantly decreased 3 months after statin treatment (Fig. 2C). While the associations of urinary and plasma GDF15 with clinical characteristics differ, as shown above, both the urinary and plasma GDF15 changed similarly in some aspects when we further investigated the association between the urinary GDF15 to creatinine ratio and other clinical characteristics in the CKD patients. For instance, both urinary and plasma GDF15 were correlated with aging to a similar extent (Fig. S3).

### Urinary GDF15 predicts the prognosis of renal function in early-stage diabetic kidney disease

**Figure 3:**
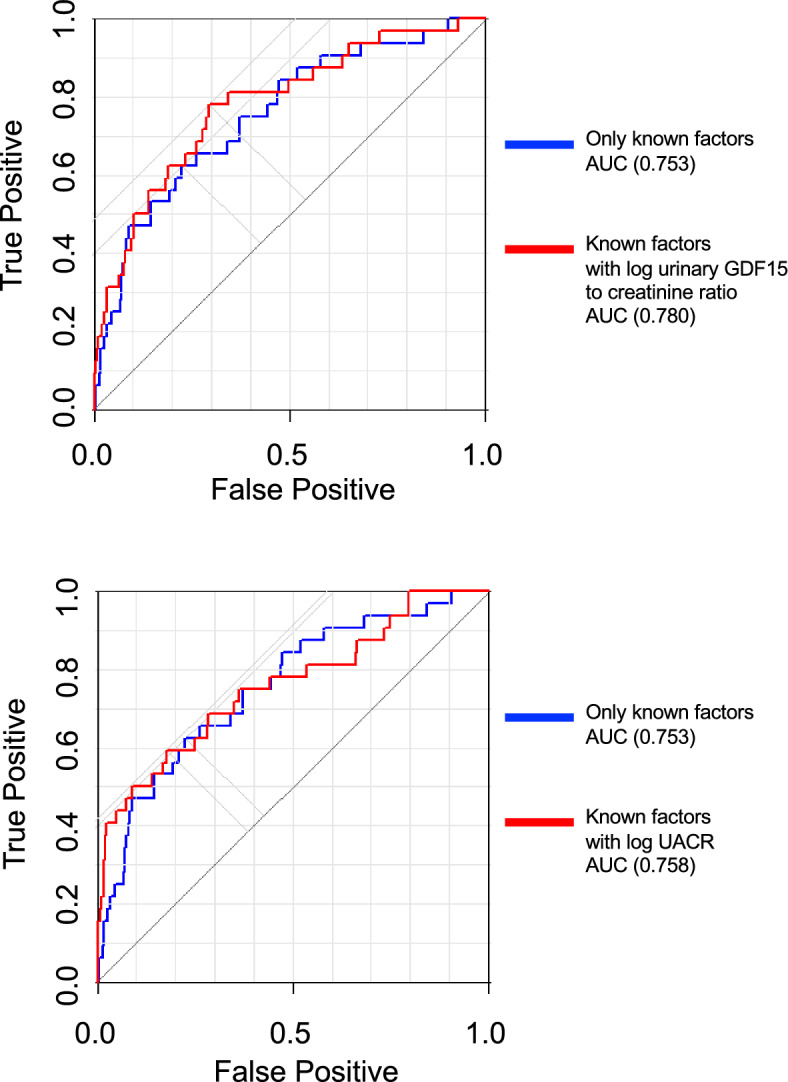
Clinical significance of urinary GDF15 in predicting decline in renal function in patients with diabetic kidney disease. Receiver operating characteristic (ROC) curve analysis in the U-CARE Study, showing a comparison of known factors^19^. (**A**) AUC-ROC using known factors (0.753) and adjusted for urinary GDF15 (0.780), (**B**) AUC-ROC using known factors (0.753) and adjusted for UACR (0.758) (n = 342). GDF15, growth differentiation factor 15; UACR, urinary albumin to creatinine ratio. Known factors: age, gender, BMI, SBP, HbA1c, and log(eGFR).

We examined whether urinary GDF15 could predict the prognosis of renal function in DKD. We recruited 342 patients with diabetes from the U-CARE cohort study (UMIN 00,011,525)^16^ (the mean age was 63.1 years, 56.7% were male, and the average eGFR was 72.3 at baseline; Table 2) and performed a retrospective cohort study. Multiple regression analysis was performed to evaluate whether the baseline urinary GDF15 to creatinine ratios would be associated with a decrease in eGFR during the 2 years. We evaluated using three models: Model 1 (crude model); Model 2 (adjusted for factors: plasma GDF15, age, sex, BMI, SBP, HbA1c, log (eGFR)); and Model 3 (full model; adjusted for Model 2 plus other clinical factors, such as DBP, ALT, TC, TG, HDL, and UA)^14^. In Model 1, the baseline urinary GDF15 to creatinine ratio was correlated with the decrease in eGFR during the 2 years. In Models 2 and 3, the urinary GDF15 to creatinine ratio was the only factor related to the decline in eGFR (*p* = 0.025 in Model 3; Table 3). Given the significant relationship between the baseline urinary GDF15 and the decline in eGFR, we further examined logistic regression models to identify factors independently involved in the decrease of renal function (Table 4). The mean follow-up duration was 2.1 ± 0.2 years. Based on Model 1, the urinary GDF15 to creatinine ratio and UACR were related to the decline in eGFR during the 2 years, suggesting that urinary GDF15 and UACR were independent risk factors for the deterioration of renal function. In Models 2 and 3, the urinary GDF15 to creatinine ratio still showed a relationship with the decline in eGFR; UACR also showed significance (urinary GDF15 to creatinine ratio: odds ratio = 1.62, *p* = 0.049, UACR: odds ratio = 1.43, *p* = 0.014 in Model 3; Table 4). While the receiver operating characteristic (ROC) curve analysis revealed that the areas under the curve of the ROC (AUC-ROC) using known factors^19^ were 0.753, the combination with the urinary GDF15 to creatinine ratio built up the c-statistic value to 0.780 (Fig. 3), and the combination with UACR increased the c-statistic value to only 0.758 (Fig. 3). These data indicated that urinary GDF15 is likely to predict the decline in eGFR in patients with DKD, similar to UACR.

**Table 2:** Baseline characteristics of 342 patients with diabetic kidney disease in the U-CARE cohort.

Number of patients	342
Demographics	
Age, y	63.1 ± 12.4
Gender, male, n (%)	194 (56.7)
BMI, kg/m^2^	24.9 ± 4.3
Medical history	
Diabetes mellitus, n (%)	342 (100)
Type 1, n (%)	40 (11.7)
Type 2, n (%)	298 (87.1)
Medications	
Antihypertensives, n (%)	178 (52.0)
ACEi/ARB, n (%)	156 (43.1)
Ca blocker, n (%)	95 (26.2)
Diuretic, n (%)	35 (9.7)
Hypoglycemics, n (%)	331 (96.8)
Insulin, n (%)	129 (35.6)
SU, n (%)	83 (22.9)
DPP4i, n (%)	186 (51.3)
Biguanide, n (%)	139 (38.4)
Thiazolidine, n (%)	44 (12.2)
αGI, n (%)	99 (27.3)
Glinide, n (%)	31 (8.6)
GLP1, n (%)	20 (5.5)
SGLT2i, n (%)	5 (1.4)
Statin, n (%)	178 (49.2)
Clinical features	
sBP, mmHg	128 ± 15.2
dBP, mmHg	72 ± 10.2
BS, mg/dL	153 ± 54.2
LDL-Chol, mg/dL	98.8 ± 25.8
Serum creatinine, mg/dL	0.82 ± 0.29
eGFR, mL/min/1.73 m^2^	72.3 ± 16.1
UACR, mg/gCre median (IQR)	11 (6.3–37.3)
GDF15 concentration	
Plasma GDF15, pg/mL, median (IQR)	1189.7 (810.2–1737.9)
Urinary GDF15, ng/mmolCre, median (IQR)	812.5 (445.6–1357.8)

BMI, body mass index; SBP, systolic blood pressure; eGFR, estimated glomerular filtration rate; DBP, diastolic blood pressure; LDL, low-density lipoprotein; UACR, urine albumin-creatinine ratio; GDF15, growth differentiation factor 15.

**Table 3:** Multiple regression analysis (objective value: the decreasing value of eGFR until 2 years).

	Model 1				Model 2				Model 3			
	Regression coefficient	95%CI		p	Regression coefficient	95%CI		p	Regression coefficient	95%CI		p
log(urinary GDF15/creatinine ratio)	− 0.0150	− 0.0277	− 0.0023	0.0210	− 0.0163	− 0.0295	− 0.0030	0.0159	− 0.0152	− 0.0285	− 0.0018	0.0259
Age					0.0001	− 0.0011	0.0013	0.8891	0.0000	− 0.0013	0.0013	0.9913
Gender					0.0052	− 0.0190	0.0293	0.6756	0.0069	− 0.0190	0.0329	0.6012
BMI					0.0011	− 0.0019	0.0040	0.4785	0.0008	− 0.0026	0.0041	0.6479
SBP					− 0.0004	− 0.0012	0.0004	0.3309	− 0.0002	− 0.0012	0.0008	0.6764
HbA1c					0.0070	− 0.0044	0.0185	0.2294	0.0076	− 0.0044	0.0196	0.2152
log(eGFR)					0.0181	− 0.0332	0.0694	0.4890	0.0163	− 0.0390	0.0716	0.5638
Duration									− 0.0009	− 0.0025	0.0007	0.2669
DBP									− 0.0007	− 0.0022	0.0007	0.3323
ALT									− 0.0003	− 0.0014	0.0008	0.6009
TC									0.0004	− 0.0001	0.0009	0.0900
TG									0.0000	− 0.0002	0.0001	0.6062
HDL									− 0.0006	− 0.0016	0.0004	0.2393
UA									0.0014	− 0.0067	0.0095	0.7367

Multiple regression analysis based on clinical factors as independent variables examined by a variance inflation factor (VIF) < 10. Urinary GDF15 was used as an independent variable. Model 1, crude model; Model 2, adjusted for known factors: age, gender, BMI, SBP, HbA1c, log (eGFR))^19^; and Model 3, full model, adjusted for Model 2 factors plus fundamental clinical data, including DBP, ALT, TC, TG, HDL, and UA) were used.

*p < 0.05 was considered statistically significant.

GDF15, growth differentiation factor 15; BMI, body mass index; SBP, systolic blood pressure; eGFR, estimated glomerular filtration rate; DBP, diastolic blood pressure; ALT, alanine aminotransferase; TC, total cholesterol; TG, triglyceride; HDL, high-density lipoprotein; UA, uric acid; CI, confidence interval.

**Table 4:** Logistic regression analysis of the association between eGFR deterioration and urinary GDF15.

	Model 1				Model 2				Model 3			
	Odds	95%CI		p	Odds	95%CI		p	Odds	95%CI		p
log(urinary GDF15/creatinine ratio)	1.7657	1.1537	2.7025	0.0088	1.6152	1.0220	2.5526	0.0400	1.6217	1.0008	2.6279	0.0496
log(urinary albumin/creatinine ratio)	1.7349	1.3866	2.1708	0.0000	1.4526	1.1109	1.8994	0.0064	1.4360	1.0751	1.9180	0.0143
Age					1.0506	1.0005	1.1032	0.0475	1.0808	1.0203	1.1449	0.0082
Gender					1.4406	0.6402	3.2414	0.3776	0.6429	0.2646	1.5618	0.3293
BMI					1.0737	0.9725	1.1854	0.1594	1.0841	0.9636	1.2197	0.1792
SBP					0.9935	0.9670	1.0207	0.6360	0.9817	0.9490	1.0155	0.2836
HbA1c					0.8233	0.5304	1.2780	0.3862	0.8478	0.5288	1.3592	0.4929
log(eGFR)					0.2510	0.0654	0.9629	0.0439	0.1128	0.0204	0.6228	0.0123
Duration									0.9678	0.9169	1.0216	0.2362
DBP									1.0452	0.9954	1.0976	0.0760
ALT									1.0144	0.9755	1.0549	0.4730
TC									0.9883	0.9696	1.0074	0.2278
TG									1.0015	0.9952	1.0078	0.6385
HDL									1.0279	0.9899	1.0673	0.1526
UA									0.9339	0.6414	1.3600	0.7215

2-year eGFR deterioration (15% reduction in eGFR over 2 years) shown by logistic regression analysis based on patients with diabetic kidney disease in the U-CARE cohort. Model 1: only log (urinary GDF15/creatinine ratio) and log UACR (crude model). Model 2: Model 1 with known factors (age, gender, BMI, SBP, HbA1c, and log eGFR)^19^. Model 3: Model 2 with other factors (duration of DM, DBP, ALT, TC, TG, HDL, and UA). The 95% confidence intervals (95% CI) are listed.

GDF15, growth differentiation factor 15; BMI, body mass index; SBP, systolic blood pressure; eGFR, estimated glomerular filtration rate; DBP, diastolic blood pressure; ALT, alanine aminotransferase; TC, total cholesterol; TG, triglyceride; HDL, high-density lipoprotein; UA, uric acid; CI, confidence interval.

## Discussion

This study showed that the urinary GDF15 to creatinine ratio was less affected by eGFR and likely to be a good prognostic marker for DKD. We recently showed that plasma GDF15 can serve as a diagnostic marker for mitochondrial diseases by reflecting the mitochondrial function in the tissue^5^. CKD progression would be associated with the mitochondrial dysfunction as well^23^. Although the precise mechanism remains unclear, mitochondrial dysfunction showing morphological change, bioenergetic impairment, and the production of reactive oxygen species (ROS) exists in DKD^24^. GDF15 could then be a good diagnostic marker for kidney diseases. However, plasma GDF15 might not be a useful marker because its level is affected by the deterioration of renal function^25^. Few studies have reported on urinary GDF15; Perez-Gomez et al. recently showed that the urinary GDF15 levels were associated with the kidney histology patterns, mortality, and the need for renal replacement therapy^12^. While their study focused on patients suffering from modest renal failure (average eGFR, 51.7), our study showed that urinary GDF15 is a useful prognostic marker for renal function in early-stage DKD (average eGFR, 73.8).

In this study, the urinary GDF15 to creatinine ratio and UACR could both predict the decline in eGFR in DKD patients. However, some studies showed that the mitochondrial dysfunction, such as an alteration of the mitochondrial mortality, bioenergetics, and ROS production, all appear prior to the presence of albuminuria^26^. Based on our data, while both the urinary GDF15 to creatinine ratio and UACR could significantly predict the decline in eGFR, the urinary GDF15 to creatinine ratio built up the c-statistic value of the ROC curve stronger than UACR (urinary GDF15 to creatinine ratio: 0.780 versus UACR: 0.758) despite a statistically non-significant difference. The urinary GDF15 might predict the decline of kidney function in DKD earlier than UACR, although further investigations are needed. Moreover, because the urinary GDF15 is produced mainly by renal tubular cells^11,12^, while albumin leakage is from the glomerulus and tubular cells, each marker might have its own advantages. Further research can be expected to show further the usefulness of urinary GDF15 as a marker for kidney diseases.

In this study, the association of urinary GDF15 and plasma GDF15 with renal function, uremic toxins, and aging varied. The differences and similarities in dynamics between plasma and urinary GDF15 may suggest various meanings in medications and pathophysiology. Statins alleviate kidney injury in non-end-stage CKD^22^. Our findings indicated that statin therapy may alleviate intra-renal damage, thereby resulting in decreased urinary GDF15 levels, especially in DKD patients. On the other hand, metformin, an anti-diabetic drug, has been reported to increase plasma GDF15 levels, thereby causing food intake reduction and body weight loss^27^. Although plasma GDF15 is a biomarker for various diseases, such as cardiovascular diseases^28^, its function has also been highlighted recently. Further studies are needed to clarify whether urinary GDF15 is associated with other pathological conditions such as senescence, cardiovascular disease, diabetes, and neurodegenerative diseases, all of which are known to cause mitochondrial dysfunction. The function of GDF15 in the kidney should be also investigated further.

In conclusion, this study revealed that the urinary GDF15 to creatinine ratio was significantly increased in patients with diabetes and turned out to be a sensitive prognostic marker for renal function in patients with DKD. The urinary GDF15 levels were less associated with renal function and the concentration of uremic toxins compared to the plasma GDF15 levels, which highlights the usefulness of urinary GDF15.

## Supplementary Information

Supplementary Figures.

Supplementary Table S1.

## Data Availability

The data in this article are available from the corresponding author upon reasonable request.

## References

[1] Colhoun HM, Marcovecchio ML (2018). Biomarkers of diabetic kidney disease. Diabetologia.

[2] Said SM, Nasr SH (2016). Silent diabetic nephropathy. Kidney Int..

[3] Lopez-Giacoman S, Madero M (2015). Biomarkers in chronic kidney disease, from kidney function to kidney damage. World J. Nephrol..

[4] Sauriasari R, Safitri DD, Azmi NU (2021). Current updates on protein as biomarkers for diabetic kidney disease: A systematic review. Ther. Adv. Endocrinol. Metab..

[5] Matsuhashi T (2017). Mitochonic acid 5 (MA-5) facilitates ATP synthase oligomerization and cell survival in various mitochondrial diseases. EBioMedicine.

[6] Adela R, Banerjee SK (2015). GDF-15 as a target and biomarker for diabetes and cardiovascular diseases: A translational prospective. J. Diabetes Res..

[7] Conte M (2022). GDF15, an emerging key player in human aging. Ageing Res. Rev..

[8] Rochette L, Zeller M, Cottin Y, Vergely C (2020). Insights into mechanisms of GDF15 and receptor GFRAL: Therapeutic targets. Trends Endocrinol. Metab..

[9] Myhre PL (2020). Growth differentiation factor 15 provides prognostic information superior to established cardiovascular and inflammatory biomarkers in unselected patients hospitalized with COVID-19. Circulation.

[10] Kim JS (2019). Association between plasma levels of growth differentiation factor-15 and renal function in the elderly: Korean Frailty and Aging Cohort Study. Kidney Blood Press. Res..

[11] Nair V (2017). Growth differentiation factor-15 and risk of CKD progression. J. Am. Soc. Nephrol..

[12] Perez-Gomez MV (2021). Urinary Growth Differentiation Factor-15 (GDF15) levels as a biomarker of adverse outcomes and biopsy findings in chronic kidney disease. J. Nephrol..

[13] Oikawa Y (2020). Mitochondrial dysfunction underlying sporadic inclusion body myositis is ameliorated by the mitochondrial homing drug MA-5. PLoS ONE.

[14] Kikuchi K (2019). Gut microbiome-derived phenyl sulfate contributes to albuminuria in diabetic kidney disease. Nat. Commun..

[15] Mishima E (2014). Conformational change in transfer RNA is an early indicator of acute cellular damage. J. Am. Soc. Nephrol..

[16] Mise K (2018). Identification of novel urinary biomarkers for predicting renal prognosis in patients with type 2 diabetes by glycan profiling in a multicenter prospective cohort study: U-CARE study 1. Diabetes Care.

[17] Coresh J (2014). Decline in estimated glomerular filtration rate and subsequent risk of end-stage renal disease and mortality. JAMA.

[18] García-Carro C (2021). How to assess diabetic kidney disease progression? From albuminuria to GFR. J. Clin. Med..

[19] Hoshino J (2018). A new pathological scoring system by the Japanese classification to predict renal outcome in diabetic nephropathy. PLoS ONE.

[20] Toyohara T (2009). SLCO4C1 transporter eliminates uremic toxins and attenuates hypertension and renal inflammation. J. Am. Soc. Nephrol..

[21] Toyohara T (2010). Metabolomic profiling of uremic solutes in CKD patients. Hypertens. Res..

[22] Liu JC, Hsu YP, Wu SY (2016). Statins and renin angiotensin system inhibitors dose-dependently protect hypertensive patients against dialysis risk. PLoS ONE.

[23] Galvan DL, Green NH, Danesh FR (2017). The hallmarks of mitochondrial dysfunction in chronic kidney disease. Kidney Int..

[24] Galvan DL, Mise K, Danesh FR (2021). Mitochondrial regulation of diabetic kidney disease. Front. Med..

[25] Yazawa H (2020). Association of serum growth differentiation factor-15 with eGFR and hemoglobin in healthy older females. Int. J. Cardiol. Heart Vasc..

[26] Coughlan MT (2016). Mapping time-course mitochondrial adaptations in the kidney in experimental diabetes. Clin. Sci..

[27] Coll AP (2019). GDF15 mediates the effects of metformin on body weight and energy balance. Nature.

[28] Wollert KC (2017). Growth differentiation factor 15 as a biomarker in cardiovascular disease. Clin. Chem..

