# Supplementary material for: Urinary growth differentiation factor 15 predicts renal function decline in diabetic kidney disease
Source: Sci Rep. 2023 Aug 2;13:12508. doi: 10.1038/s41598-023-39657-7 (PMC10397309; doi:10.1038/s41598-023-39657-7)
Supplement: Supplementary file 1 — Supplementary Figures. [file 41598_2023_39657_MOESM1_ESM.pdf]

Fig. S1

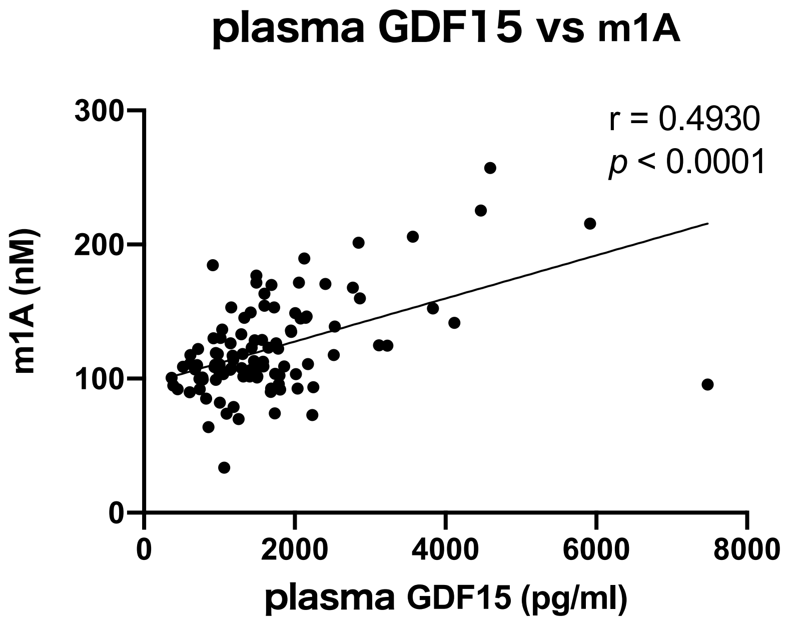

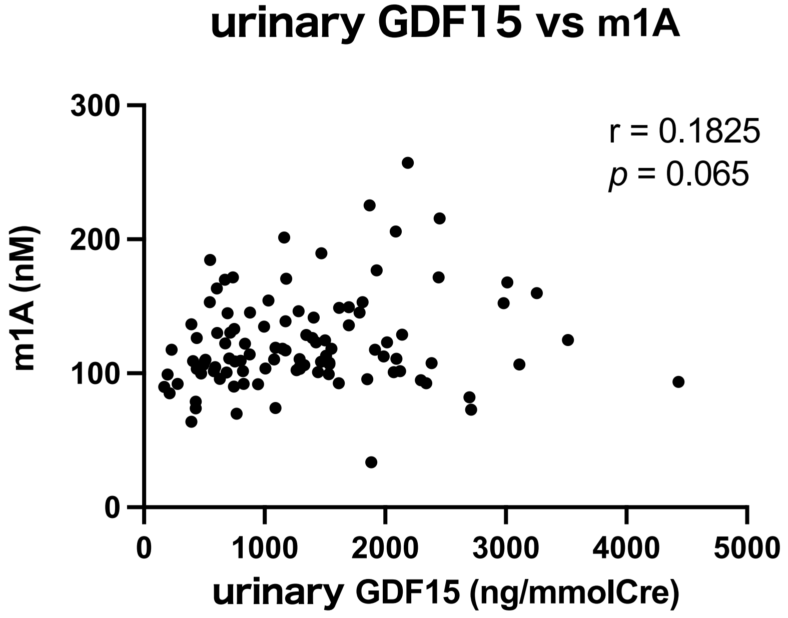

Figure S1. Correlations of plasma and urinary growth differentiation factor 15 (GDF15) with 1-methyladenosine (m1A). The Spearman correlation coefficient was used to assess the relationships. $p < 0.05$ was considered statistically significant.

Fig. S2

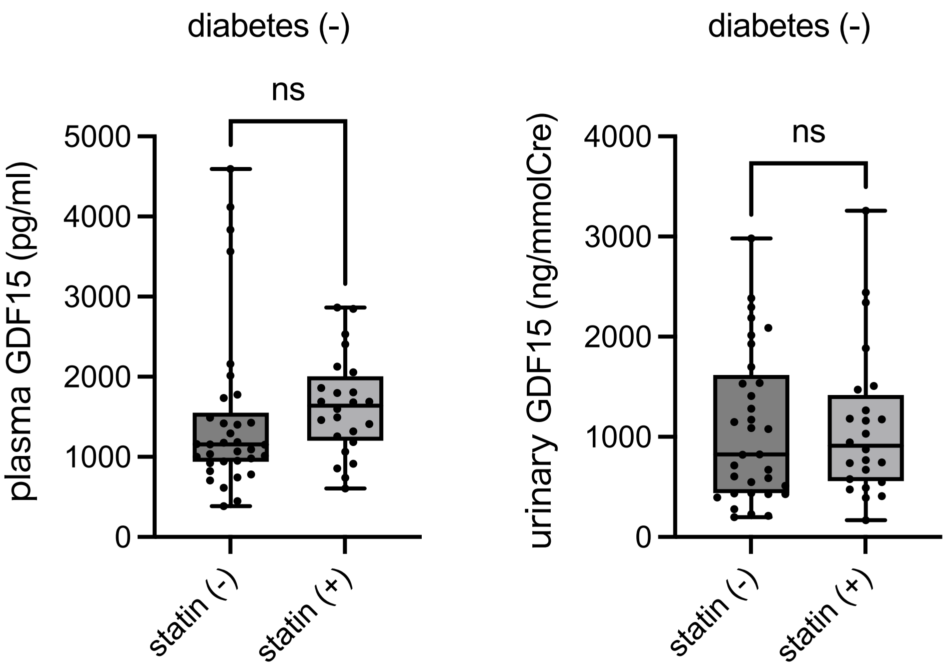

Figure S2. Concentration of plasma and urinary GDF15 in CKD patients without diabetes receiving statin treatment or not (n = 24 and 33, respectively). An unpaired t-test was used to compare two groups.

Fig. S3

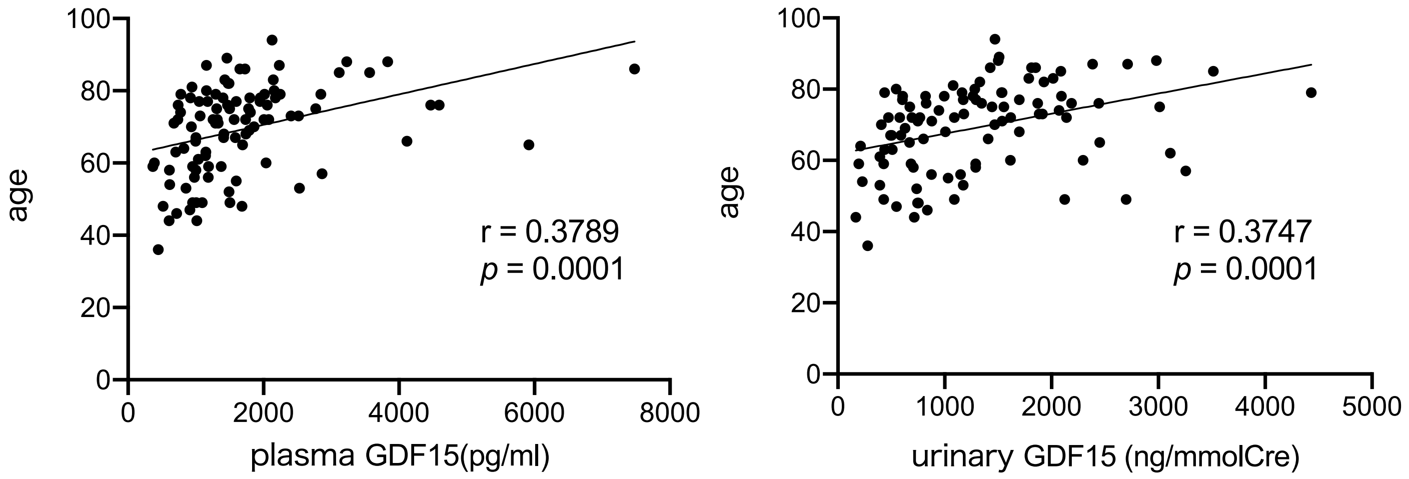

Figure S3. Correlations of plasma and urinary growth differentiation factor 15 (GDF15) with age. The Spearman correlation coefficient was used to assess the relationships. $p < 0.05$ was considered statistically significant.
