# Supplementary material for: Urinary growth differentiation factor 15 predicts renal function decline in diabetic kidney disease
Source: Sci Rep. 2023 Aug 2;13:12508. doi: 10.1038/s41598-023-39657-7 (PMC10397309; doi:10.1038/s41598-023-39657-7)
Supplement: Supplementary file 2 — Supplementary Table S1. [file 41598_2023_39657_MOESM2_ESM.pdf]

Table S1. Baseline characteristics of 39 patients with statin treatment.

Number of patients	39
Demographics	
Age, y	62.1 ± 12.1
Gender, male, n (%)	25 (64.1)
BMI, kg/m²	23.6 ± 4.2
Medical history	
Hypertention, n (%)	38 (97.4)
Diabetes mellitus, n (%)	10 (25.6)
Medications	
Antihypertensives, n (%)	38 (97.4)
Hypoglycemics, n (%)	7 (18.0)
Clinical features	
sBP, mmHg	131.2 ± 9.9
dBP, mmHg	75.2 ± 6.0
Serum creatinine, mg/dL	1.64 ± 0.66
eGFR, mL/min/1.73 m²	41.5 ± 18.1

SBP, systolic blood pressure; DBP, diastolic blood pressure; eGFR, estimated glomerular filtration rate.
